# Brain-Age Prediction Using Shallow Machine Learning: Predictive Analytics Competition 2019

**DOI:** 10.3389/fpsyt.2020.604478

**Published:** 2020-12-02

**Authors:** Pedro F. Da Costa, Jessica Dafflon, Walter H. L. Pinaya

**Affiliations:** ^1^Centre for Neuroimaging Sciences, Institute of Psychiatry, Psychology & Neuroscience, King's College London, London, United Kingdom; ^2^Centre for Brain and Cognitive Development, Birkbeck College, London, United Kingdom; ^3^Department of Biomedical Engineering, King's College London, London, United Kingdom

**Keywords:** brain-age, shallow machine learning, linear models, genetic algorithm, support vector machine

## Abstract

As we age, our brain structure changes and our cognitive capabilities decline. Although brain aging is universal, rates of brain aging differ markedly, which can be associated with pathological mechanism of psychiatric and neurological diseases. Predictive models have been applied to neuroimaging data to learn patterns associated with this variability and develop a neuroimaging biomarker of the brain condition. Aiming to stimulate the development of more accurate brain-age predictors, the Predictive Analytics Competition (PAC) 2019 provided a challenge that included a dataset of 2,640 participants. Here, we present our approach which placed between the top 10 of the challenge. We developed an ensemble of shallow machine learning methods (e.g., Support Vector Regression and Decision Tree-based regressors) that combined voxel-based and surface-based morphometric data. We used normalized brain volume maps (i.e., gray matter, white matter, or both) and features of cortical regions and anatomical structures, like cortical thickness, volume, and mean curvature. In order to fine-tune the hyperparameters of the machine learning methods, we combined the use of genetic algorithms and grid search. Our ensemble had a mean absolute error of 3.7597 years on the competition, showing the potential that shallow methods still have in predicting brain-age.

## 1. Introduction

As we age, our brain manifests cognitive decline (1, 2) and several structural changes, such as cortical thinning, reductions in brain volume, and decline in white matter microstructure (3–5). Although brain aging is universal, differences between individuals rates of brain aging can be substantial. In some cases, these differences can characterize clinically relevant deviations of psychiatric and neurological diseases (6, 7).

Recently, studies have been using machine learning methods to predict the brain age of individuals. This task is performed by modeling trajectories and patterns of brain aging of a healthy population. Most of these studies are based on structural Magnetic Resonance Imaging (MRI), where researchers have been trying to map structural features [e.g., regional volume, thickness, and mean curvature; (8–11)], and volume maps [i.e., gray and white matter or a combination of both; (12–14)], to the chronological age of the subjects. In order to analyse the effect of diseases in the brain aging rate, researchers have been training machine learning models on healthy subjects and using the trained model to perform predictions on patient's data. The difference between the predicted age and chronological age is thought to be a marker for the individual's risk of developing any age-associated disease or cognitive decline. Based on this line of thought, several neurological and psychiatric diseases have been showing findings of pathological mechanisms that manifest as accelerated aging; for example, major depressive disorder (15), multiple sclerosis (16, 17), Alzheimer's disease (18), schizophrenia (19).

Although Relevance vector Regression (RVR), Support Vector Regression (SVR), Gaussian Process Regression are the most commonly used methods to predict brain age (8, 9, 13, 20), recently other methods such as Convolutional Neural Networks have gained popularity (13, 21). Unfortunately, because on the current literature, it is hard to disentangle if the differences in performance of the algorithms are due to the differences in sample size and characteristics from the dataset or due to the algorithm's performance.

Aiming to stimulate the development of more accurate brain-age predictors, the Predictive Analytics Competition (PAC) 2019 provided a challenge that included a big dataset of 2,640 healthy participants. In this study, we combined several types of shallow machine learning methods [i.e., conventional machine learning models that in contrast to deep learning models are not characterized by multiple processing layers; (22)] to predict the brain age of the subjects from the PAC 2019. In our approach, we used genetic-based methods and grid search to tune the hyperparameters of the models. We also incorporated information from different structural features, such as regional features (i.e., volume, thickness, and mean curvature), gray matter, and white matter normalized volume maps, as well as, information about the acquisition sites in order to improve the performance of our predictions. We hypothesized that an ensemble of shallow methods could offer competitive results in this competition.

## 2. Methods

**Figure 1:**
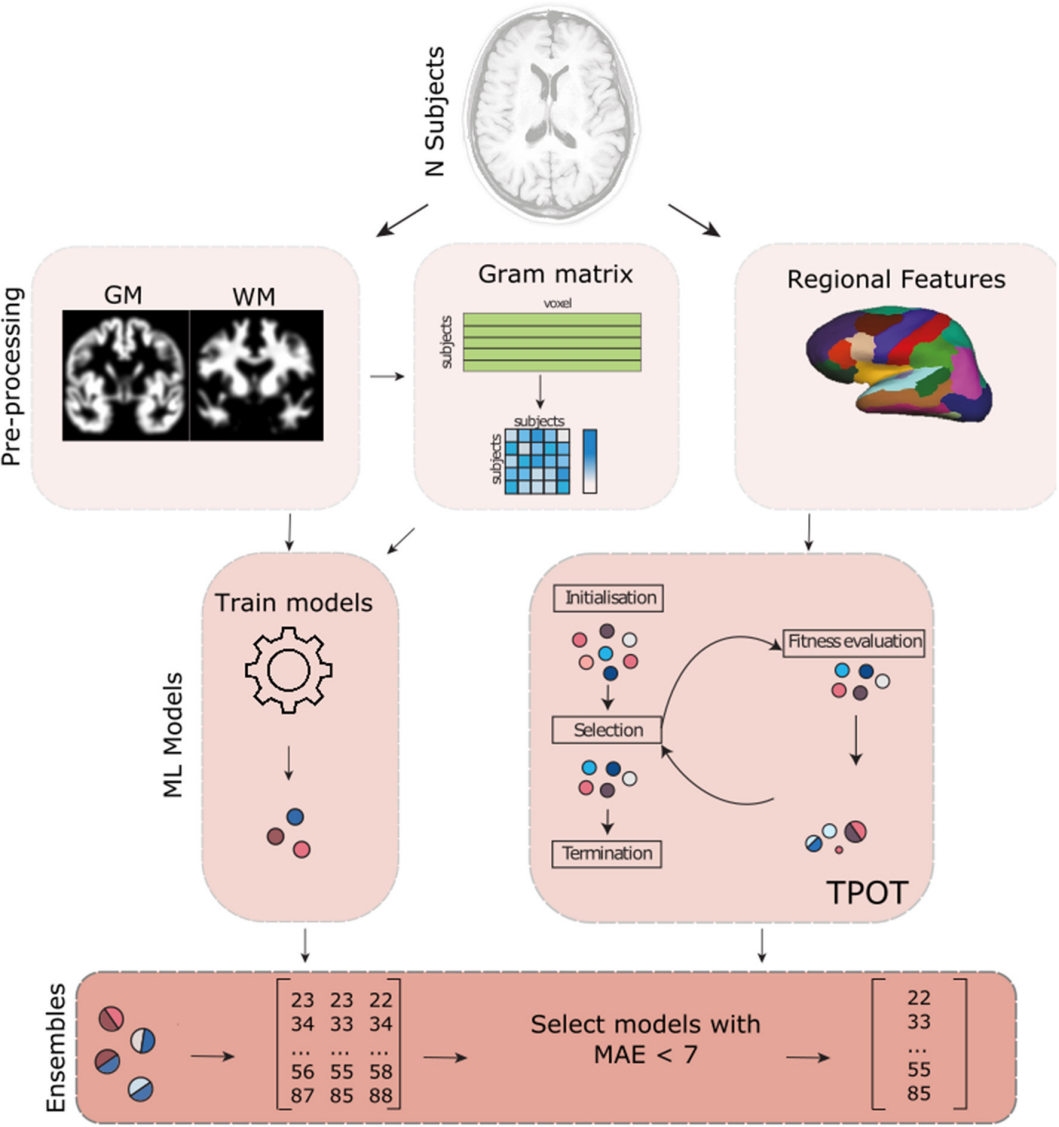
Overview of the different methods used in our analysis. In addition to the gray matter (GM) and white matter (WM) volume maps provided by the PAC competition, we also pre-processed the data in order to obtain the regional volume, thickness and mean curvature information of the brain using Freesurfer. We then used different strategies that involved creating a gram matrix, dimensionality reduction algorithms (e.g., PCA) and TPOT (an automated machine learning framework) to train different models. In addition, to using different pre-processing, we also trained different models for the different sites where the data was recorded. All models that had a mean absolute error (MAE) lower than 7 years were used to build a weighted ensemble.

See Figure 1 for an overview of the methods used. All code used for the analyses is available on GitHub (https://github.com/Mind-the-Pineapple/mind-the-gap).

### 2.1. Dataset

The data used in this analysis were derived from T1-weighted MRI images. All participants of the competition were provided with the raw NIfTI files as well as the pre-processed data (13). This dataset was acquired in 17 different sites that were not disclosed. The cohort used for training our algorithms consisted of *N* = 2, 640 healthy individuals (male/female = 1, 237/1, 403, mean age = 35.87±16.20, range 17−90). An independent test set (*N* = 660) was used to validate the performance of the model submitted by each participating team.

### 2.2. Pre-processing

#### 2.2.1. Normalized Brain Volume Maps

The pre-processed normalized volume maps were already provided by the PAC 2019 organizers and were generated following the process described in (13). Briefly, this method consisted in segmenting gray matter (GM) and white matter (WM) volumetric maps using SPM12 (University College London, London, UK) according to their tissue classification. The normalization to the MNI152 was performed using DARTEL and a 4 mm Gaussian smoothing kernel. The size of the smoothing kernel was chosen to be the default value, which has been commonly used in previous research (12, 23). Lancaster et al. (24) explored the impact of voxel size and kernel size and observed that the values suggested by using Bayesian optimization are close to the values commonly used. To facilitate comprehension and inform the reader, we have briefly reported here how the WM and GM extraction was performed, however, we did not perform this step during our analysis. The only pre-processing that we have applied to the data was the FreeSurfer analysis, which has been described in the section below. We have used the WM and GM volumes that were provided by the PAC organizers. For our analysis we used a combination of GM, WM, and GM+WM maps as input for our models. All maps were acquired using all voxels and data were pre-processed in order to ensure that all images were brought into the same space for the appropriate machine learning analysis.

#### 2.2.2. Brain Regional Features

We also extracted structural features using a surface-based approach implemented by FreeSurfer pipeline (v6.0). We obtained the estimations of the cortical thickness, volume, and mean curvature and anatomical structure volumes using the “*recon-all”* command [more detailed information about the processing in (25, 26)]. The cortical surface of each hemisphere was parcelated according to the Desikan-Killiany atlas (27). This process calculated the cortical thickness, volume, and mean curvature for each of the 68 brain regions (34 in each hemisphere) and volumes of the 45 anatomical structures (saved as stats/aseg.stats under the FreeSurfer subject directory).

### 2.3. Shallow Machine Learning Algorithms

Brain age has been a focus of research in the past few years, resulting in a rich literature on the topic (13, 28). Despite this, there is little agreement on which model performs best on brain data to predict age, mainly due to wide variations in methodologies and types of data. There are three classical machine learning models that are commonly used to predict brain age: Linear Regressors (LR), Support Vector Regressors (SVR), and Gaussian Process Regressors (GPR). Because of their popularity, in this work we trained these three models to predict brain age on the different types of data pre-processing previously described using K-fold cross-validation on the training set. The very large number of features made it computationally unfeasible to train the model directly on the brain volume maps. To overcome this limitation the pair-wise kernel matrix was pre-computed to reduce the dataset to an *NxN* matrix, where *N* refers to the number of data points, and was passed to the SVR models. As for the linear regressor model, the number of features in the dataset was reduced by Principal Component Analysis (PCA), by preserving 95% of the original variance of the dataset. This allowed to reduce the dimensions used by the models while still maintaining most of information. Besides training on the whole dataset, the models were also trained separately on each individual site, to adjust for the known problem of between-scanner variability (29). The main idea behind this is that by training all sites separately, every model will only learn biological features that are relevant to predict brain age and non-biological information (i.e., different scanner settings) or potential dataset biases cannot be learned by the model.

#### 2.3.1. Linear Regression

LR is a simple parametric modeling approach that tries to model the relationship between the independent variables, *X*, and the target variable, *y*. It does so, by adapting the weights θ to fit a linear equation to the observed data. This modeling of the data has an analytical solution to obtain the optimal θ (Equation 1).

LR assumes that the relationship between the independent variables and the target variable is linear, which is a drawback from this model, as the brain data that serves as input is highly non-linear regarding the dependent variable, age (30). The main advantages of this modeling approach are its simplicity, transparency, and analytical solution (Equation A1).

#### 2.3.2. Support Vector Regressor

SVR is a supervised learning model that fits a regression to the training data by minimizing the distance of the sampled points to a margin of tolerance around the fitted hyperplane (31). This is a sparse algorithm, which means it only requires the information of a small number of data points (i.e., support vectors) to define the hyperplane that is used for prediction of unseen data. This facilitates handling of datasets with a high number of data points. We mapped the original space into a kernel space by applying a pair-wise kernel function. By pre-computing the kernel space, we greatly reduce the computational resources spent training the model, as the number of variables is reduced to be the same size as the number of data points. We obtained the regularization hyperparameter *C* by using Grid-search over the search space of {2^−6^, 2^−5^, 2^−4^, 2^−3^, 2^−2^, 2^−1^, 2^0^, 2^1^}. The hyperparameter *C* is used to reduce overfitting by virtue of a trade-off between the regression complexity and the precision of the model.

#### 2.3.3. Gaussian Process Regressor

GPR is a non-parametric modeling approach that uses Bayesian inference to solve regression problems (32). It does so, by learning a probability distribution of possible target values based on a Gaussian process (GP) prior, which incorporates the prior knowledge of the space. The GP is specified using a mean function, 0 in this case, and a covariance function also called kernel. In this work we analyzed three different kernels: a pre-computed pair-wise kernel, Radial Basis Function kernel (RBF) and a white kernel. Because this modeling approach is not sparse, unlike the SVR, it is computationally burdensome, especially when dealing with high number of variables as is the case in voxel-based data. Due to resource limitations, the GPR was only trained on the surface-based morphometry data.

### 2.4. TPOT Models

TPOT [Tree-based Pipeline Optimization tool; (33), https://zenodo.org/record/3872281] is an open source framework that uses genetic programming to test multiple pipelines and find the most appropriated machine learning model for the problem at hand.

TPOT allows the user to define a pool of algorithms to be used. This pool of models can contain models that are pre-defined by TPOT or can include any model written by the user, or even be from any available package [e.g., the scikit-learn library; (34)] chosen by the user. The pool of models is not limited to machine learning algorithms but can include different pre-processing as well feature transformations algorithms. For this analysis we have included to the pool of models available to TPOT, not only the most popular algorithms to predict brain age (e.g., SVR, RVR, and GPR), but we also include other linear models (e.g., Lasso and Ridge Regression). To see a full list of the models used the reader can refer to Supplementary Table 1.

TPOT works by (i) selecting the algorithms from the user defined pool of algorithms, (ii) using with a cross-validation approach it trains those chosen algorithms and pass those with the highest accuracy to the next generations, (iii) the 20 pipelines with the best performance will be mutated/cross-over and passed to the next generation, (iv) once the last generation is reached (the number of generations is specified by the user before starting the analysis) the model with the highest accuracy and lowest complexity will be returned to the user. Therefore, together with the fact that TPOT allows the best model and parameter to be chosen in a data-driven fashion, one of its main advantages is that it penalizes overfitting by selecting the pipeline with the best performance but the lowest number of algorithms.

### 2.5. Ensemble

Ensembles of models tend to outperform single models and are a common technique to bolster algorithm's accuracy (35, 36). Ensembles tend to be more flexible in the functions they can represent, as they are not limited to a single hypothesis space of each model it is composed from. To perform a weighted ensemble, we used the mean absolute error (*MAE*) of each model on the K-fold cross validation. All models with a *MAE* above 7, our baseline, were discarded. The weight, *w*, given to a model's prediction was calculated by the squared difference between the baseline and the obtained MAE, in order to benefit smaller errors, following Equation 2. The weights were then used for a weighted average of the final prediction. For each site, only models trained on the site and on the whole training set were considered (Equation A2).

## 3. Results

The aim of our study was to develop pipelines that precisely predict the subject's age. To do this, we divided our analysis into two parts (i) we trained our pipelines using the data from all sites, (ii) we trained separate models for the 17 different sites. In addition, because different features might be more important for specific models, we also explored the effects of the different structural and regional features.

### 3.1. All Sites Analysis

Among the most used models to predict brain age, the SVR model trained with a combination of GM and WM achieved the best performance (MAE = 4.571 years; Table 1). These results are in line of those observed by (13) where they describe an increase in performance by combining both GM and WM information.

**Table 1:** Performance of each machine learning model when using the whole dataset.

**Model**	**Data type**	**MAE**
SVR	WM data	5.589
SVR	GM data	5.004
SVR	GM+WM data	4.571
SVR	vol	7.187
LR	PC from GM data	13.609
LR	PC from WM data	13.613
GPR	curv	7.200
GPR	thk+vol	6.385
GPR	thk+vol+curv	6.132

*The results are presented as the mean MAE for a 5-fold cross validation. WM, white matter volumetric map; GM, gray matter volumetric map; vol, regional volume; curv, regional mean curvature; thk, regional thickness; PC, principal components*.

On the other hand, when using TPOT and the regional features to find the most appropriate model, the returned pipeline consisted of a combination of linear regression and random forest regressor and yielded a MAE of 5.195 years.

### 3.2. Different Models for the Different Sites

To avoid non-biological variability induced by the different scanners, acquisition protocols and field strengths, we trained our best performing model from Table 1 (i.e., the SVR model which combined GM + WM information) using the data from each site separately. The performance of the site-specific models is reported in Table 2. The big oscillation in the MAE can be attributed to the difference in demographics of the different sites. As expected, the age-range per site correlated positively with the models' MAE (mean Pearson correlation coefficient across sites ± sd: 0.710 ± 0.001), as sites where participants had a small range of ages were easier to predict. Sample size per site had little correlation with the models' MAE (0.117 ± 0.656) and so did the sex ratio (0.147 ± 0.573).

**Table 2:** Performance of the SVR model when using White Matter + Gray Matter volumetric data from each specific site.

**Site #**	**MAE**
0	5.087
1	4.473
2	4.887
3	3.620
4	1.662
5	4.527
6	3.091
7	9.777
8	3.850
9	5.678
10	6.266
11	5.188
12	4.846
13	7.084
14	7.070
15	1.159
16	2.447

*The results are presented as the mean MAE for a 3-fold cross validation*.

Similarly, we also used the regional features to train TPOT in a site-specific fashion. In contrast to the results presented in Table 2 where we only used an SVR model and compared it's performance among the different sites, here we allowed TPOT to search for the best pipeline for each individual site (Table 3; to improve the readability here we only presented the models that composed the pipeline. If the reader is interested to know the models and their hyperparameters that lead to the optimal performance please see our Github—https://github.com/Mind-the-Pineapple/mind-the-gap). Interestingly, although none of the sites had the same pipeline the site-specific performance was in general comparable to that obtained when using only the SVR.

**Table 3:** Performance of the resulting TPOT pipelines when using thickness, volume, and mean curvature information from each specific site separately.

**Site #**	**Pipeline**	**MAE**
0	3 Lasso + RVR + Ridge + RF	5.557
1	Lasso + KNR	4.101
2	ElasticNet + Extra Trees + Ridge	4.721
3	Linear SVR + RF	4.027
4	2 Extra Trees + Ridge	2.05
5	RF	6.667
6	2 GPR	5.940
7	2 ElasticNet	5.638
8	ElasticNet + RF	3.938
9	Lasso + RF + Extra Trees	6.685
10	KNR + DT + Ridge	9.210
11	RVR	4.213
12	DT + Ridge	4.375
13	2 RF + DT + Ridge	10.155
14	Extra Trees + 2 DT + LR + Ridge	10.849
15	LR	1.861
16	RF + ElasticNet + DT	2.220

*The results are presented as the mean MAE for a 5-fold cross validation. Lasso, lasso model fit with least angle regression; RVR, relevance vector regressor; Ridge, linear least squares with l2 regularization; RF, random forest; KNR, K-neighbors regressor; DT, decision tree; GPR, gaussian process regressor; LR, linear regression*.

Finally, we combined the predictions of our models with a MAE <7 into an ensemble. To make sure that weakly performing models would not negatively impact our ensemble performance, we weighted the model's prediction on the ensemble based on its performance. In this weighted combination, we verified that none of the trained linear regressions performed well enough (their MAE was bigger than 7 years) to be included in the ensemble analysis, therefore we excluded any linear regression model from the ensemble. Models trained on individual sites were only considered for ensembles predicting data from their respective site. As different sites might have different scanners and other non-biological variations, by training each site separately every model learns the features that are relevant for brain-age prediction and its individual scanner properties and by keeping the sites independently it allows us to better account for inter-scanner variability. A crucial limitation that derives from this design choice is that the site information needs to be released together with the dataset. As this was the case for the PAC competition, we could use the subject's site information to choose the best model to predict brain age for that individual. Our ensemble had a mean absolute error of 3.7597 years on the independent test set, which was used to evaluate the performance of the different teams of the PAC 2019. To put this result into perspective, the best model, which consisted of an ensemble of computational intensive deep-learning models (37), achieved a performance of 2.9043 years on the same dataset.

## 4. Discussion

In this paper, we showed that shallow machine learning methods yield competitive results when predicting the brain age of the subjects from the PAC 2019 competition. In our approach, we used genetic-based methods and grid search to tune the hyperparameters of the different shallow models and trained the models using different structural measures. Importantly, we also trained different models for the different sites so that we could better account for scanner variability.

Deep learning's popularity is extending widely in various areas of research and is becoming a common tool in neuroscience. However, it is still an open question if brain images can profit from deep neural networks to learn the non-linearities from brain images with the current small datasets and a high number of features, while still being able to generalize to unseen datasets (21, 38, 39). This discussion arises from the fact that neural networks require more observations in order to learn complex patterns and significantly surpass the performance of classical shallow methods. Besides, if deep learning methods are not provided with sufficient data, they will be more prone to overfit and not generalize due to the large number of parameters in the models. To illustrate this problem, while ImageNet, one of the most commonly used datasets to train deep-learning models to classify natural images, contains about 14 million images, the UK Biobank, one of the biggest research consortia, currently provides 45,000 brain scans and aims to have 100,000 by 2050 (40).

Given that the dataset provided by the competition consisted of a large number of participants (N >1, 000), our results support the findings from (21). They showed that while for two benchmark datasets used in machine learning (i.e., MNIST and Zalando Fashion datasets) the performance of the deep-learning methods improved with an increase in the number of samples used to train the methods, that was not the case for linear models, where a plateau performance was reached. For neuroimaging datasets (i.e., volumes, connectivity, and slices) the performance of shallow models did not approach a plateau and had very similar performance as deep-learning models. Therefore, this suggests that even by using a larger dataset, the maximal performance of shallow models are not reached when using neuroimaging datasets. Similarly, He et al. (39) showed that kernel methods are as precise as neural networks when predicting behavior but have a lower computational cost. Some other noteworthy advantages of linear models and shallower models compared to deep neural networks are: (i) they are in general easier to interpret (22); (ii) they are less computationally intensive and can more quickly be trained, (iii) deep learning architectures are hard to adapt to the problem at interest, therefore, one of the biggest limitations of deep learning is to adapt previous architecture to the problem at hand. An appropriate adaptation requires vast experience from the practitioner; (iv) linear models can run in any computer and does not require GPU access.

One of the biggest challenges of machine learning is to find the appropriate hyperparameters for the model to be trained (41). Due to the large number of possible models, their hyperparameters and suitability for the problem at hand, finding the most appropriate combination can be a bewildering and computational intensive task. To address this issue, in this competition we used: (i) grid search strategy, which repeatedly performed the analysis over a set of pre-defined hyperparameters; (ii) a genetic-based method that was performed by TPOT in order to find the most appropriate model and its hyperparameters (i.e., taking into account both precision and complexity). Similarly, to the results reported by Dafflon et al. (42) and the *no free lunch principle* (43), we observed that there was not a single model that always had the best performance when predicting age (Table 3). The different models identified by TPOT for each site probably changed due to biological (i.e., age range, population heterogeneity) and non-biological factors (i.e., field strength and scanner manufacturer). As previous studies reported, these confounding variables have a significant influence on the performance of machine learning applications in neuroimaging data (44–46). Nevertheless, the combination of models suggested by TPOT leads to an improved performance that probably balances the strength and limitations of the single models by combining them into a pipeline. Another interesting feature of TPOT is that while searching for the *best* model, TPOT penalizes models that obtain a better performance due to overfitting. Despite the risk of overfitting of some of our site specific pipelines, due to the small sample size of some sites, the out-of-sample evaluation performed by the PAC committee with an independent dataset revealed a good performance.

In this paper, we have also taken into account the scanner where each data point originated from, building site-specific models, before combining them with models trained on all scanners. This was an effort to address the common issue of data variability between scanners, which can add variability in the dataset (47). For example, different scanner manufacturers, field strengths, or acquisition protocols which might have an effect on the algorithm's performance. One limitation of this site-specific approach is that some scanners have a small number of participants, resulting in models trained with low number of data points. To avoid overfitting to the sites, we discarded the models with poor performance (MAE>7). Another limitation of predicting age from brain images is the inter-variability and heterogeneity (i.e., different degrees of brain aging that might reflect different life styles, genetics, exclusion/inclusion criteria, and undiagnosed diseases) even in healthy participants, resulting in a noteworthy irreducible error in brain age prediction. In line with this idea, Holmes and Patrick (48) proposed that variability is also present in healthy controls and should be better addressed.

In conclusion, this paper shows that leveraging shallow models and ensemble learning to predict age from brain data is a simple but effective way of obtaining successful predictive models, despite the intrinsic non-linearity of the data. This approach also results in more interpretable models than deep learning models, as it is easier to deconstruct the model's mechanisms. Ultimately, this simple approach obtained a top-10 qualification in the PAC 2019 competition, competing directly with more complex and non-linear predictive models.

## Data Availability Statement

The data analyzed in this study is subject to the following licenses/restrictions: The dataset is part of the Predictive Analytics Competition (PAC) 2019. Requests to access these datasets should be directed to Tim Hahn, Hahn_T@klinik.uni-wuerzburg.de.

## Author Contributions

PD, JD, and WP designed and performed the experiments and wrote the manuscript. All authors contributed to the article and approved the submitted version.

## Conflict of Interest

The authors declare that the research was conducted in the absence of any commercial or financial relationships that could be construed as a potential conflict of interest.

## Appendix

$$\begin{array}{l} \theta ={\left(X^{T}\cdot X\right)}^{-1}\cdot X^{T}\cdot y\;\left(A1\right) \end{array}$$

$$\begin{array}{l} w_{model}={\left(7-MAE_{model}\right)}^{2}\;\left(A2\right) \end{array}$$
