# Supplementary material for: Brain-Age Prediction Using Shallow Machine Learning: Predictive Analytics Competition 2019
Source: Front Psychiatry. 2020 Dec 2;11:604478. doi: 10.3389/fpsyt.2020.604478 (PMC7738323; doi:10.3389/fpsyt.2020.604478)
Supplement: Supplementary file 1 [file Table_1.pdf]

frontiers

Supplementary Material

Algorithms	Sklearn Implementation
Principle Component Analysis	PCA
Fast algorithm for Independent Component Analysis	FastICA
Select the p-values corresponding to Family-wise error rate	SelectFwe
Select features according to a percentile of the highest scores	SelectPercentile
Remove low-variance Features	VarianceThreshold
Agglomerate features	FeatureAgglomeration
Elastic Net model with iterative fitting along a regularisation path	ElasticNetCV
Randomised Decision Trees on sub-samples of the dataset	ExtraTreesRegressor
k-Nearest Neighbours Regression	KNeighborsRegressor
Cross-validated Lasso using the LARS algorithm	LassoLarsCV
Linear Support Vector Regression	LinearSVR
Linear Least squares with l2 regularisation	Ridge
Gaussian process regression	GaussianProcessRegressors
Random Forest Regressor	RandomForrestRegressor
Ordinary Least Squares Linear Regression	LinearRegression
Decision Tree Regressor	DecisionTreeRegressor
Relevance Vector Regression	RVR∗

Table S1. List of models available for TPOT.
∗ : The RVR algorithm is not part of the Sklearn library. The algorithm used here was provided https://github.com/JamesRitchie/scikit-rvm
